# Biomaterials for Pelvic Floor Reconstructive Surgery: How Can We Do Better?

**DOI:** 10.1155/2015/968087

**Published:** 2015-04-21

**Authors:** Giulia Gigliobianco, Sabiniano Roman Regueros, Nadir I. Osman, Julio Bissoli, Anthony J. Bullock, Chris R. Chapple, Sheila MacNeil

**Affiliations:** ^1^Material Science Engineering, University of Sheffield, Sheffield S1 3JD, UK; ^2^Royal Hallamshire Hospital, Sheffield S10 2JF, UK; ^3^Hospital das Clínicas da Faculdade de Medicina, Universidade de Sao Paulo, 05410-020 São Paulo, Brazil

## Abstract

Stress urinary incontinence (SUI) and pelvic organ prolapse (POP) are major health issues that detrimentally impact the quality of life of millions of women worldwide. Surgical repair is an effective and durable treatment for both conditions. Over the past two decades there has been a trend to enforce or reinforce repairs with synthetic and biological materials. The determinants of surgical outcome are many, encompassing the physical and mechanical properties of the material used, and individual immune responses, as well surgical and constitutional factors. Of the current biomaterials in use none represents an ideal. Biomaterials that induce limited inflammatory response followed by constructive remodelling appear to have more long term success than biomaterials that induce chronic inflammation, fibrosis and encapsulation. In this review we draw upon published animal and human studies to characterize the changes biomaterials undergo after implantation and the typical host responses, placing these in the context of clinical outcomes.

## 1. Introduction

Stress urinary incontinence (SUI) and pelvic organ prolapse (POP) are important health problems that cause a sizable personal, societal, and economic burden [1]. SUI is defined as the “involuntary leakage of urine on exertion, sneezing or coughing” [2, 3]. POP is the “the descent of one or more of the anterior vaginal wall, posterior vaginal wall, the uterus (cervix), or the apex of the vagina (vaginal vault or cuff scar after hysterectomy)” [4]. SUI and POP are thought to share a common pathogenesis, weakening of the muscular and connective tissues of the pelvic floor. Multiple etiological factors have been implicated including ageing, obesity, pregnancy, and childbirth, as well as genetic factors and menopause [1, 5–7].

Following failure of conservative management including physiotherapy, corrective surgery is considered to be the most effective and durable treatment for both SUI and POP. Most of the older surgical techniques relied upon suturing the local tissues to the back of the pubic bone (colposuspension) or using an autologous fascial sling. More recently there has been a growing trend to reinforce repairs using both synthetic and biological materials. This practice has been adapted from hernia surgery where there is established evidence that repairs reinforced with synthetic mesh provide superior outcomes.

Synthetic meshes were popularized in pelvic floor surgery for SUI following the work of Ulmsten and Petros [8]. The mid-urethral tape (MUT) involved a minimally invasive approach to implant a thin synthetic mesh underneath the mid-urethral point. Early reports of cure rates in the range of 80–90% further propelled the uptake of this technology. Following the early success of MUT and a randomized control trial against colposuspension, synthetic mesh for SUI was soon introduced [9]. This was not based on long term supportive data but rather a grandfather clause which permitted introduction of a new material based on its similarity to an index product, which was used for hernia repair, namely, polypropylene mesh. A long term follow-up, the Ward and Hilton [9] study, demonstrated a 4% exposure of mesh rate. Subsequently mesh was introduced for the treatment of pelvic organ prolapse (POP) and this has resulted in a significant problem with mesh exposure which has led to enormous medico-legal problems, particularly in the United States of America.

The following decade has seen a rapid rise in reports of mesh for POP related complications, but it is clearly important to differentiate mesh exposure (erosion) used for SUI from that used for POP. Thus reports of debilitating complications of vaginal mesh implantation have emerged including vaginal wall erosion (0–25.6%), chronic pain (0–5.5%), and sexual problems (1.9–17%) [10]. Although it can be debated whether these rates are high, the complications are often difficult to treat, requiring further hospital visits, further tests, and further reconstructive surgery. The situation has not escaped the attention of medical regulatory bodies such as the FDA who have issued statements warning patients and surgeons of the potential dangers of mesh use for POP [11, 12]. More recently there has been a wave of class action litigation law suits raised against device manufacturers by patients who have suffered mesh complications, such that several major manufacturers have withdrawn products from the market.

Biological grafts are alternatives to synthetic mesh. The most commonly used material, autologous fascia, has been used for over 100 years in the treatment of SUI with good efficacy. The main drawback however is the need to harvest the graft from a donor site (fascia lata from the thigh or rectus fascia from the abdominal wall) and potential morbidity (e.g., wound infection, scar, nerve injury, and hernia) [13]. There is a limitation on how much graft can be harvested which precludes its use in POP which is associated with relatively large fascial defects. This can be avoided by using grafts derived from cadavers or alternatively animal derived collagen matrices (e.g., porcine dermis, porcine small intestine, and bovine dermis). However, these materials require extensive processing decellularization, sterilization, and cross-linking processes to resist degradation [14]. While this renders materials nonimmunogenic, it can impact their biomechanical properties [15]. There is also the risk of viral or prion transmission [13]. Clinical studies are limited; however clinical experience is that all of the materials appear to be associated with graft failure in the medium term due to the body's response to the material, leading its encapsulation and subsequent degradation with limited remodeling.

It is likely that biomaterials are subject to multifactorial problems because of (1) their physical properties (e.g., porosity and degradability), (2) their mechanical properties (e.g., stiffness and strength), or (3) the nature of the patient's immune response to the implanted biomaterials. In addition, surgical and patient specific factors (e.g., individual anatomy and comorbidities) are likely to play a role, though these are not modifiable by material design.

**Figure 1:**
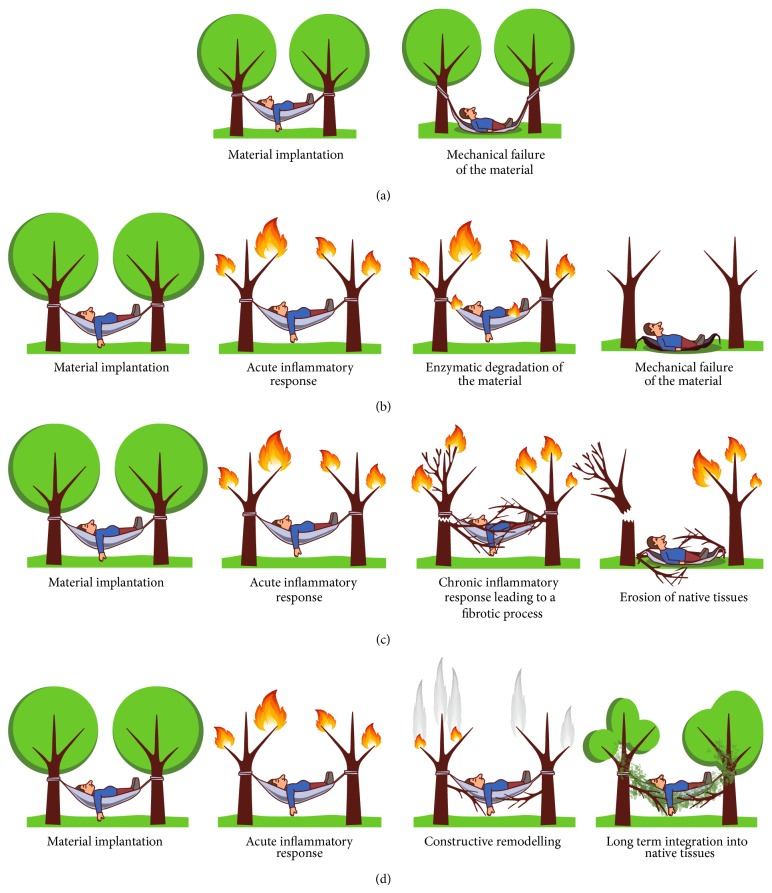
Cartoon of how patients can respond to materials implanted in the pelvic floor: (a) mechanical failure, (b) material recognized as non-self and isolated from body tissues with encapsulation, (c) exposure (erosion), and (d) optimal result for implanted material.

To provide a simple context for this review we depict the current hypotheses of how failures of implant might occur through several routes in cartoon form in Figure 1 where the implanted material is shown conceptually as a hammock attached to two trees (the supporting structures of the pelvic floor).

In the case of successful implantation, it is currently thought that the material induces an acute inflammatory response, which leads to constructive remodeling and material integration (Figure 1(d)).

The aim of this review is to characterize these changes and responses, from the available human and animal studies, and relate them to clinical outcomes, thereby guiding the design of novel materials for this challenging clinical application.

## 2. Methods

The MEDLINE database was searched for articles describing studies investigating the* in vivo* response to biomaterials used routinely in pelvic floor surgery or that have been studied in the context of clinical trials. The search was limited to the years 1990 to 2013. The following search terms were used: “pelvis,” “pelvic floor,” “vagina,” “*in vivo*,” “*in vitro*,” “biocompatibility,” “prolapse,” “incontinence,” “biomaterial,” “sling,” “mesh,” “polypropylene,” “autografts,” “allografts,” and “xenografts.” Abstracts were screened for relevance by 2 reviewers before full articles were retrieved. Articles were included if they described the changes in physical or biomechanical properties of materials after implantation in animals or humans or the histological features of the host response to the implanted material. Implantation sites were restricted to subcutaneous, intravaginal, or abdominal muscles.

## 3. Results

In total 10 studies assessing autologous materials, 11 assessing allograft materials, 24 assessing xenografts, and 24 assessing polypropylene meshes compared with other synthetic meshes were included. These studies are summarized in Tables 2, 3, 4, and 5.

### 3.1. Biological Materials

#### 3.1.1. Autologous Materials

Autologous grafts harvested from the rectus fascia and fascia lata have long been used in SUI surgery. A major advantage of autografts over synthetic materials is that erosion is almost unheard of [16]. A possible disadvantage to using autografts is that the connective tissues of patients with SUI may be inherently weak predisposing to failure. Nevertheless the overall long term outcomes with autografts are largely excellent with reported rates of cure generally over 90% [17, 18].

*Biomechanical Properties of Autologous Materials.* Four studies describing changes in mechanical properties of autologous materials over a 12–16-week period were found. Uniaxial stress strain testing of autologous rectus fascia before and after implantation in rabbit vagina and anterior abdominal wall showed no significant decrease of ultimate tensile strength (UTS) (the maximum stress a material can take before failing) and Young's modulus (YM) (material stiffness), at twelve weeks after implantation [19, 20]. However, there was a reduction in surface area of the grafts by 50% suggesting that significant degradation had occurred [19, 20]. A comparison of mechanical strength of autologous materials used for sling was carried out by Choe et al. [21]. They harvested dermis, rectus fascia, and vaginal mucosa from 20 women undergoing vagina prolapse surgery and they tested displacement and maximum load with the Instron tensiometer. This study showed that fascia lata had the highest mean maximum load to failure (217 N), followed by human dermis (122 N), rectus fascia, and vaginal mucosa (both 42 N) in women undergoing surgeries for various reasons [21]. Autologous rectus fascia showed no significant decrease in tear resistance using the trouser tear test after 4 months of subcutaneous implantation in rodents [22]. In summary in all four studies there was agreement that the mechanical properties did not change significantly over a 12- to 16-week duration [19–22]. 

*Host Response to Implanted Autologous Materials.* Eight studies analysed the host response to autologous materials over a time period up to 90 days [19, 20, 23–29]. In the majority of studies, unless stated otherwise, biomaterials were assessed after implantation by conventional blindly scored histology (staining of fixed samples by haematoxylin and eosin (H&E)), trichromes, and/or the presence of proangiogenic cells.

Hilger and colleagues assessed human cadaveric skin and autologous fascia after implantation in the abdominal and vaginal walls of New Zealand white rabbits. Materials were harvested at 6 and 12 weeks. Histological analysis demonstrated that autologous fascia promoted a relatively minimal inflammatory response and neovascularization but moderate collagen infiltration when compared to fenestrated porcine dermis and porcine collagen-coated polypropylene mesh [20]. Jeong and coworkers described similar results noting minimal inflammatory response and neovascularization in rabbits when autologous fascia was implanted under the eye lid for up to 8 weeks [24].

Two studies assessed histological changes in paravaginal tissue after the implantation of autologous fascial slings for SUI in women. In the study by FitzGerald et al. biopsies of the sling were taken from 5 patients requiring revision surgery due to persistent incontinence. The time since the initial surgery ranged from 3 weeks to 4 years. The grafts explanted after up to 8 weeks showed moderate uniform fibroblast infiltration and neovascularization. Collagen remodelling was evident in parts of the graft biopsied at 4 years, with no evidence of chronic inflammation [23]. Woodruff and colleagues performed a similar study in 24 patients undergoing sling revision for poor efficacy (2 patients), urinary retention (9), and sling obstruction (13), 2–34 months after implantation [27]. All grafts showed moderate uniform fibroblast infiltration and moderate collagen fibers. All grafts showed moderate degradation. There was no evidence of encapsulation.

In summary these eight studies suggest that when autologous fascia is implanted there is a minimal to moderate inflammatory response, a moderate degree of collagen production, and a suggestion that grafts undergo a degree of remodelling over the long term.

#### 3.1.2. Allografts

Allografts used in pelvic floor reconstruction usually consist of fascia. The donors are screened for infectious diseases before the grafts undergo cleaning, freeze drying, and gamma irradiation to eradicate any infective or immunogenic material. A concern with these grafts is that they are often donated by the elderly who have an age related weakening in connective tissues [30]; additionally processing techniques such as freeze drying and solvent dehydration may reduce the tensile strength [31]. Cadaveric grafts are advantageous in that they avoid donor site complications. In terms of efficacy, results are mixed. Some have shown cadaveric fascia to demonstrate similar subjective cure rates to autologous fascia at around 90% at 2 years [32]. However others have shown that on urodynamic testing 42% of cadaveric graft patients had SUI whereas no patients with autologous grafts had SUI [33]. 

*Biomechanical Properties of Allografts.* Five studies investigated the change in mechanical properties after implantation of allografts in animals. All these studies utilized uniaxial stress strain testing. The time after which samples were explanted ranged from 60 days to 12 weeks [20, 22, 34–36].

After implanting human cadaveric dermis in rabbit vagina, Hilger et al. reported a decrease in ultimate strength of 86.6% at 12 weeks; in comparison autologous fascia lost only 28.6% [20]. Conversely, Rice and colleagues found an increase in tensile strength of cadaveric dermis (AlloDerm) from 0.142 to 0.226 MPa, increasing by about 80% of its initial strength, 60 days following subcutaneous implantation [36]. Walter et al. reported that, after 12 weeks, following implantation of cadaveric fascia lata in rabbit vagina, the tensile strength decreased by approximately 90% [34]. Spiess et al. implanted human cadaveric fascia lata subcutaneously on the abdominal wall of 20 rats randomized into 2 survival groups at 6 and 12 weeks. They found no significant decrease in tensile strength from 0.167 kg at week 6 and 0.185 kg at week 12 [35]. Kim et al., similarly, implanted human cadaveric fascia in 20 rats, randomized into 2 survival groups of 2 and 4 months. They found no significant difference in fracture toughness before implantation and after implantation in human cadaveric fascia (from 2120 to 1145 J/m^2^, *P* = 0.09) [22].

In summary, the available studies show disparate results with respect to the changes in mechanical properties of allografts following implantation. This may be attributable to the heterogeneity in the type of allografts used, the animals studied, the sites of implantation, and the assessment at different time points.

*Host Response to Implanted Allografts.* In total eight studies assessed the host response to allografts in both animals and humans. The time since implantation ranged from 2 days up to 65 weeks [20, 26, 27, 36–40].

Human cadaveric dermis and cadaveric fascia have been found to be well integrated onto the abdominal wall [37, 40, 41] and rectus muscle [36, 38] in different animals, including rats, rabbits, and pigs, as noted by moderate fibroblast infiltration, new collagen production, and neovascularization where materials were implanted from 2 days up to 62 weeks. Human cadaveric dermis, after 12 weeks of implantation, was similarly well integrated into vaginal tissues of rabbits. However, it appeared highly fragmented suggesting significant degradation [20]. Krambeck et al. also describe a faster degradation of cadaveric fascia implanted subcutaneously on the abdominal wall of rabbits with a fascial defect for 6 and 12 weeks compared to polypropylene or autologous fascia [26]. VandeVord and colleagues also found moderate cell infiltration and angiogenesis at 12 weeks following the insertion of human cadaveric dermis and cadaveric fascia slings under the bladder neck of rats; however there was a moderate encapsulation after implantation [39]. Finally, in the study by Woodruff et al. in 5 women who received human cadaveric dermis grafts, biopsies 2–65 months after implantation showed significant graft degradation with residual areas of graft appearing acellular and encapsulated [27].

In summary, some studies suggest that allografts demonstrate infiltration by host cells, new collagen production, and neovascularization whilst other studies suggest that a variable degree of graft degradation occurs along with encapsulation in the long term. There is a degree of agreement that allograft induces an acute inflammatory response as inflammatory infiltrates have been found populating the grafts.

#### 3.1.3. Xenografts

A number of grafts from animals, mainly porcine and bovine, have been used in pelvic floor surgery. These materials undergo extensive processing after harvesting to decellularize them and render them non-immunogenic. Additionally there are FDA regulations on animal source and vaccination status which must comply with [42]. Porcine dermis may be artificially cross-linked using hexamethylene diisocyanate to make it more resistant to enzymatic digestion [43]. Clinical studies showed lower continence rates for porcine dermis (approx. 80%) and increased reoperation than that for synthetic tape or autologous fascia [44]. Porcine small intestine submucosa (SIS) has shown cure rates from 79 to 93% at 2- and 4-year follow-up, respectively [45, 46]. However one study has raised concerns that SIS may not be strictly acellular and may contain porcine DNA [47].

*Biomechanical Properties of Xenografts.* Nine studies investigated the mechanical properties of xenografts before and after implantation. All these studies assessed either porcine dermal collagen matrix, both cross-linked and non-cross-linked, or porcine small intestine submucosa.

Hilger et al. assessed non-cross-linked porcine dermis xenografts implanted on the abdominal wall and vaginal wall of rabbits. After 12 weeks, half of the grafts implanted in the vaginal wall were absent. The other half as well as grafts implanted into the abdominal wall showed an average reduction of 84.1% in ultimate strength [20]. Another study assessed the long term mechanical integrity of cross-linked porcine dermis. After 9 months following implantation in the abdominal and vagina walls, grafts had degraded by 36% and 46%, respectively. When subjected to mechanical testing non-degraded graft fragments showed similar strength compared to baseline values whilst degraded fragments decreased by more than 50% [48].

Liu and colleagues implanted SIS and porcine dermal collagen matrix in rats with surgically created abdominal wall defects. The maximum load (at failure) at baseline for SIS and dermal collagen matrix was 22.81 N and 43.16 N, respectively. Following 12 weeks of implantation, there was no significant change in the maximum load of cross-linked porcine dermal collagen matrix and SIS [49]. Similarly other workers observed an increase in the ultimate tensile strength of SIS after 90 days of implantation from a baseline value of 7.5 and 9.8 N/cm^2^ at baseline, respectively, to 19.56 and 13.3 N/cm. These results were averages of 48 implants in rats [50]. Rice et al. also found an increase in tensile strength of SIS after 60 days of implantation in a rat abdominal wall defect from 0.142 MPa at day 0 up to 0.226 MPa after 60 days of implantation [36]. Similarly, Zhang et al. implanted SIS in abdominal wall of rats and they found increased strength for SIS from 0.35 MPa to 0.41 after 4 weeks [51]. Badylak et al. repaired surgically created abdominal wall defects in dogs with SIS (8 × 12 cm); they performed serial ball burst strength tests after 1, 4, 7, and 10 days and then at 1, 3, 6, and 24 months) [52]. There was an initial decrease in ball burst strength from 73.37 pounds to 39.97 pounds by day 10. After day 10, the strength began to increase and after 2 years there was an increase to 157.20 pounds in burst strength. Jenkins et al. showed an increase in strength in cross-linked porcine matrices after 6 months of implantation in the preperitoneal area from 0.07 ± 0.01 N up to 22.36 ± 3.3 N [53]. In contrast, Ko and colleagues found no significant difference in ultimate tensile strength of SIS after 4 months of implantation in a porcine wall defect, with values ranging from 41.3 to 74.8 N/cm^2^ [54].

In summary it appears that non-cross-linked porcine dermal collagen matrices are degraded rapidly (within 3 months) and lose most of their mechanical integrity within this period. By contrast cross-linked porcine dermal collagen matrix is more resistant to degradation and maintains its mechanical properties for at least 3 months, whereas SIS appears to increase in strength after as long as 2 years after implantation.

*Host Response to Implanted Xenografts.* Twenty-four studies assessing the host response to allografts were found. Non-cross-linked porcine dermal collagen was assessed in fourteen studies [20, 26, 27, 36, 39, 50–52, 54–61]. These studies were performed on rats [36, 50, 51, 55, 58, 59], dogs [52, 55], pigs [54, 57], and rabbits [26] in addition to few clinical studies [20, 27, 39, 56]. Cross-linked porcine matrices were assessed in seven studies [40, 49, 53, 62–66]. Animal models mainly used were abdominal defects of rats [49, 62, 66], rabbits [65], minipigs [53], pigs [40, 63], and primates [64]. Some of these studies looked at the acute response [39, 49, 53, 55, 59, 66]; some other studies looked at a more intermediate response (1–3 months) [20, 26, 36, 39, 40, 49–51, 54, 55, 57, 58, 60, 62–64, 66]; another looked at longer term response (more than 3-months) [27, 40, 53, 62, 64, 65].

Hilger et al. and Pierce et al. found minimal neovascularization and collagen ingrowth in porcine dermal xenografts [20, 65]. Both studies agreed that the degradation of porcine dermis is higher when the inflammatory response is high, and it may accelerate this degradation process. They also reported fragments encapsulated, which has been also found in many studies with different species including rats [39, 62], rabbits [65], pigs [40], primates [64], and humans [27].

In contrast, non-cross-linked SIS leads to high collagen ingrowth with a moderate degree of remodeling and orientation and high neovascularization [29, 36, 39, 49–51, 54, 55, 57, 63]. On the other hand, many studies agree with a very rapid degradation of the SIS which is replaced by the host tissue [49, 51, 52, 55, 58, 66, 67]. Only two studies reported an absence of host fibroblast infiltration and fibrotic tissue penetration without neovascularization for SIS implanted in rats [62] and rabbits [26]. In humans, Cole et al. performed revision surgery on a patient who had developed a bladder outlet obstruction after SIS implantation and found that the implant had been encapsulated [60]. Nevertheless, other investigators, at 12 and 48 months, respectively, found that the SIS was replaced by native tissue in humans [56, 61].

In summary, the available studies agree that the degree of cross-linkage affects the rate of degradation and the degree of the inflammatory response of the host. Studies on cross-linked xenografts agree that cross-linked collagenous matrices induce little cell infiltration; hence there is limited collagen remodeling and graft degradation. In non-cross-linked xenografts, cell infiltration was greater with faster degradation rate and collagen production.

### 3.2. Polypropylene Mesh

There is a range of synthetic polypropylene meshes that have been used. These are summarized in Table 1 where they are classified as type 1, 2, 3, or 4 according to their mesh size, where 1 is macroporous (>75 *μ*m), 2 is less than 10 *μ*m, 3 is microporous with microporous compartments, and 4 is nanoporous (<1 *μ*m). Thus a wide range of synthetic materials have been investigated for use in the treatment of SUI. These materials offer several advantages including lack of transmission of infectious diseases and ease of availability, as well as the sustainable tensile strength due to their nondegradable nature [68]. Mesh materials have been classified in to 4 groups based on the basis of porosity (microporous or macroporous) and filamentous structure (monofilament of multifilament) [69]. The initial clinical experience with mid-type II (microporous/multifilament fibers, e.g., expanded PTFE) and III (macroporous and microporous/multifilament fibers, e.g., Mersilene) meshes was largely negative with excision rates of up to 30% for expanded PTFE [70] and erosion rates of 17% for Mersilene (polyester) [71].

A greater pore size is thought to be advantageous as it allows the admittance of immune cells and greater collagen ingrowth into the construct [13]. This is thought to reduce the risk of mesh infection and accelerate and enhance host tissue integration. Monofilament meshes are thought to reduce the risk of infection in comparison to multifilament meshes. The theoretical concern with the latter is that bacteria may colonize the 10 *μ*m subspaces between fibers which are inaccessible for the larger host immune cells (9–20 *μ*m) [72]. Today a mid-type I polypropylene mesh that is macroporous and monofilament is most commonly used [73] with cure rates for SUI of >90% at 5 years.

*Biomechanical Properties of Polypropylene.* Seven studies investigated the mechanical properties of polypropylene meshes with implantation times ranging from two weeks in animal models up to two years. Animal models used were rats abdominal wall [35, 74], pig preperitoneal implantation [75], rats rectus fascia [76], minipigs hernia repair [77], and ewes abdominal and vaginal walls [78].

Melman et al. tested Bard Mesh, a knitted monofilament mesh made of high molecular weight polypropylene (HMWPP) and Ultrapro, a knitted macroporous composite mesh made of low molecular weight polypropylene (LMWPP) and poliglecaprone (Table 1). They have been implanted in minipigs hernia repair model for up to 5 months. HMWPP mesh decreased from maximal load at failure 59.3 N at 1 month to 36.0 N at 5 months, while LWPP mesh decreased from 61.5 to 37.8 N at 5 months [77]. Long term studies were carried out by Zorn et al. where TVT and SPARC were compared to SIS in a rat abdominal wall defect for up to 12 months. Both TVT and SPARC are macroporous meshes made of polypropylene monofilaments. SPARC did not change its mechanical properties after 12 months of implantation (maximum load at baseline 0.453 kg and at 12 months 0.497 kg). By contrast the maximum load for TVT decreased from 0.779 kg to 0.523 kg for TVT and for SIS decreased from 0.402 kg to 0.174 kg [74]. Also Bazi et al. showed how similar are the mechanical properties of Gynecare TVT and Advantage, both macroporous polypropylene monofilament meshes, compared with other meshes such as IVS Tunneller, multifilament polypropylene mesh, and SPARC. The lowest, at 25.2 N, was TVT and the highest, 34.9 N, was Advantage, with no significance between them after 24 weeks of implantation in rats rectus fascia [76]. Also other studies agree on these parameters where TVT was found to be able to comply with the highest break load (0.740 kg), compared to 0.39 kg for fascia lata after implantation in rats abdominal wall for up to 12 weeks [35], and was said to be less stiff than other synthetic materials used for meshes (0.23 N/mm compared to nylon, 6.83 N/mm) [79].

A recent study compared two sizes of meshes implanted in two different places in a sheep model. Gynemesh was cut in two sizes (50 × 50 mm and 35 × 35 mm) and it was implanted in 20 adult ewes, on the abdominal and vaginal walls for a period of 60 and 90 days. Results showed that grafts of both dimensions, implanted on the vaginal wall, were stiffer than the ones implanted on the abdominal wall, after a period of 90 days [78].

However, they all agree that physical characteristics of the mesh, such as monofilament or multifilament, porosity, and polymer molecular weight, hugely affect the mechanical performance of the implants* in vivo*. 

*Host Response to the Implanted Polypropylene.* Twenty-one papers have looked at the host response to the polypropylene meshes. They have been assessed in various animal models: rats abdominal wall [50, 58, 74, 80–82], rats rectus fascia [38, 76, 83], rabbits bladder neck [84], rabbits abdominal wall [85], rabbits rectus fascia [26], rabbits vaginas [65, 86], minipigs hernia [77], pigs peritoneum [75, 87], ewes vagina [78, 88], and ewes abdominal wall [78] in addition to few clinical studies [27, 89–91]. The studies have looked at acute inflammatory responses to the most commonly used, nondegradable meshes, described in Table 1. Few studies looked at the acute inflammatory response that occurs from the day of implantation up to 30 days [50, 58, 80–82, 85, 88]. Other studies looked at the immediate responses (1–3 months) [26, 75, 78, 83, 84, 86, 87] and longer term responses (>3 months) where fibrosis and chronic inflammation can be seen [27, 65, 74, 76, 77, 89–91].

A very recent study of Manodoro et al. showed how 30% of Gynemesh grafts (50 × 50 mm), implanted in ewes after 90 days, caused vaginal erosion and exposure. The study also showed that 60% of the smaller Gynemesh meshes (35 × 35 mm) had a reduced surface (i.e., contracting) after 90 days of implantation [78].

Falconer et al. reported a study on Prolene and Mersilene meshes. The biopsies were stained with Masson's trichrome. Mersilene was found to induce a higher inflammatory response compared to Prolene, which triggered a minimal inflammatory reaction [89].

Pierce et al. reported a long term study comparing biological and synthetic grafts implanted in rabbits. Polypropylene caused a milder inflammatory reaction with more long term, better host tissue incorporation compared to natural grafts [65]. Also Bazi et al. evaluated biopsies on the basis of inflammatory infiltrate, fibrosis, mast cell presence, muscular infiltration, and collagen filling of the mesh on an arbitrary scale described as low, moderate, or extensive based on H&E, periodic acid-Schiff, and toluidine blue staining of tissue. They agreed that all of the materials (Advantage, IVS, SPARC, and TVT) induced inflammation and collagen production, with SPARC being the one with the mildest response and TVT the one with the highest inflammatory response [76]. Elmer et al. reported an increase in macrophages and mast cell counts and a mild but persistent foreign body response to polypropylene meshes [91]. This study is consistent with other reported investigations where the polypropylene meshes are invaded with both macrophages and leukocytes, signs of inflammation, resulting in collagen production [27, 38, 65, 76, 83, 85].

In summary the studies agree that polypropylene meshes provoke a fairly pronounced inflammation, leading to a massive cell infiltration into the scaffold and ultimately to collagen production [27, 29, 48, 76, 83, 84, 86, 90–92].

## 4. Relating Postimplantation Changes to Clinical Outcomes

### 4.1. Biomechanics

In general, when biological materials fail this is due to enzymatic degradation after implantation, leading to a loss of mechanical support and weakening of the repair. This appears to apply particularly to the non-cross-linked xenogenic matrices. Chemically cross-linking appears to prevent this degradation and improve the mechanical outcomes. Unfortunately there is a lack of clinical evidence on how these mechanical outcomes translate into patient outcomes. Autologous grafts are the most successful biological material used in contemporary practice and the studies reviewed appear to support the long term mechanical integrity of these grafts. Nevertheless, they present several important limitations that are related to the need to harvest from a donor site. However use of cadaveric tissues avoids these limitations; however their quality depends on the age and comorbidities of the donor and this is maybe the reason for the mixed results in mechanical properties. This is consistent with the available clinical studies which suggest that allografts have poorer cure rates than autologous grafts.

We have found that polypropylene maintains its morphology and strength after implantation for up to 24 weeks [35, 74, 76]. However there was evidence that stiffness increases [77, 93]. This is consistent with durable cure rates particularly in SUI surgery (there is still some question regarding efficacy of transvaginal POP repair, compared with native tissue repair). The major issue with polypropylene meshes is the associated serious complications, in particular vaginal or urinary tract exposure (up to 10–14%). There is some evidence that meshes with greater stiffness cause the surrounding tissue to weaken, an effect termed stress shielding [94]. This can be compared to the effect of metal implants on the surrounding bone after orthopedic surgery. This effect could lead to thinning of the surrounding vaginal tissues as predisposing to erosion.

### 4.2. Host Response

Biomaterials implanted into the body will always attract the attention of the immune system. With some materials there is an M1 macrophage response of constructive remodeling; this appears to be the case with some biological matrices, SIS in particular. With materials which the body cannot remodel or integrate such as polypropylene meshes, the macrophage response is much more aggressive, an M2 macrophage response [95, 96].

It appears that a state of constant inflammation can be generated by some patients in response to some of these nondegradable materials. Constant inflammation leads to an upregulation of degradative enzymes; although these enzymes cannot degrade the material, they may damage the surrounding extracellular matrix and contribute to tissue thinning and mesh exposure. Moreover perpetuation of the inflammatory response can also result in activated fibroblasts, which produce excessive collagen laid down in a disorganized fashion around the implant (i.e., fibrosis), encapsulating the material. A small amount of fibrosis is arguably advantageous to the repair in SUI, providing a stable back stop allowing urethral compression. However excessive fibrosis may lead to mesh contraction resulting in increased pull on the adjacent tissues leading to complications such as voiding dysfunction, pain, and painful intercourse. In POP this excessive fibrotic response can lead to mesh exposure which presents a major reconstructive surgical challenge, often necessitating repeat procedures with no guarantee of symptom resolution. Nevertheless with the observation that the vast majority of patients do well with mesh, it can be concluded that some degree of fibrosis is helpful to the surgical management whereas clearly excessive fibrosis is detrimental.

Implantation of autologous fascia in general showed good integration within host tissues, associated with a low inflammatory response, compared to polypropylene meshes and degree of graft remodelling in the available human studies [50, 84]. It must be borne in mind that the human studies were all reoperative cases for clinical failure. It is difficult to speculate on whether all successful outcomes result in fully integrated and remodelled graft. Non-cross-linked xenografts are associated with clinical failure due to rapid degradation which is presumably too soon for the regeneration of strong tissues in its place [20, 24, 29]. The cross-linked grafts avoid this but rather similar to the synthetic mesh are associated with a perpetuated inflammatory response as the body is unable to integrate and remodel them. This ultimately leads to encapsulation of the graft. It would therefore seem appropriate that there should be a proper balance of degradation and replacement by new host tissue with xenografts. SIS appears to fulfill this.

This relationship between grafts and host tissues will vary for different materials and with different individuals. Here it is worth noting that as many as 15% of the population are allergic to nickel and more than 80% can become sensitized to nickel on sustained exposure [97] and that there are very successful studies involving muscle regeneration using decellularized ECM [98]. Therefore, it is clear that the immune response to any foreign material is complex, dynamic, and patient specific. The fact that polypropylene meshes provoke little adverse reaction when implanted in the abdominal wall for hernia repair but are associated with complications in the pelvic floor may also suggest a site-specific host response notwithstanding the differences in biomechanical aspects [99]. This contrasting response has been confirmed in ewes [78], therefore the need for relevant animal models for longer studies [100].

## 5. Perspective on the Ideal Material

Whilst authors have previously described paradigms of the ideal material, we suggest that these have been unrealistic [101]. Ultimately a permanent material will always cause complications in some patients due to variation in individual immune responses. Conversely degradable materials will fail in some patients. The question is which is least desirable? Whilst recurrent symptoms can always be treated by corrective surgery, the complications of polypropylene mesh such as chronic pain have proven resistant to treatment in many cases. Thus we suggest that materials for this application should be degradable based on the principle of least harm. With this in mind, it is essential that the degradability is tuned so that it allows enough time for the development of a neotissue that is able to mechanically support the pelvic organs. A material that does not cause any inflammation is unrealistic and probably undesirable as an initial inflammatory response is required to promote angiogenesis and collagen ingrowth, integrating the material. This is essentially an M1 macrophage response. For this to happen, the material should be readily permeable to host cells. On a practical level any material for this application needs to be robust to withstand surgical handling and provide support at the point of insertion. We suggest that a more realistic material for this application would be the one that

- is degradable,
- provokes an acute inflammatory response,
- undergoes tissue remodeling,
- is permeable to cells,
- is mechanically robust at point of implantation.

## 6. Conclusion and Future Perspective

The clinical experience suggests that both synthetic and biological materials can provide successful outcomes when used in the surgical management of pelvic floor disorders. However, it has become clear that there is an incidence of significant complications of polypropylene meshes and that many surgeons do not consider the complication rate acceptable. Both the host response and the mechanical properties of the materials need to be taken into consideration to predict success of the implants, in addition to their response to dynamic loading. There has clearly been a lack of adequate preclinical evaluation with polypropylene mesh and we suggest several steps which may make the development for new materials an altogether safer endeavor:

- a better understanding of the forces within the pelvic floor, whose materials need to cope with when implanted;
- computational modeling of how materials might perform under load for many years (this can be achieved using* in virtuo* models once established);
- the investigation of immune responses in patients in whom materials perform well over many years versus patients in whom they cause severe complications (using biochemical markers, genomic markers, and non-invasive imaging);
- the development of better animal models that develop the complications associated with vaginal mesh use such as exposure;
- establishment of standardized criteria to evaluate the performance of materials in* in vivo* and* in vitro* studies so that they can be accurately compared.

There are several other factors which require urgent attention but are beyond the scope of this review. Surgical expertise based on training and experience in reconstructive surgery is a key factor in outcomes of pelvic floor procedures and there is a need to ensure that surgeons are adequately trained. Patient specific issues, such as individual anatomy and tissue strength, could also impact outcomes and further investigation remains necessary to assess these aspects and their role in determining outcome [102]. Although databases to track complication rates exist, such as MAUDE and Postmarket Surveillance Studies, the medical community needs to participate more fully in these databases in order to more critically audit patient outcomes and move forward.

Ultimately to develop new effective and safe materials there is a need for a multidisciplinary approach that combines the efforts of those working in regenerative medicine, biomaterials, and surgery.

## Figures and Tables

**Table 1:** Classification of synthetic materials used in pelvic floor reconstruction.

Type	Mesh pore size	Structure	Polymer	Trade name	Company
I	Macroporous >75 *μ*m	Monofilament	Polypropylene	Uretex	C. R. Bard
				Gynecare TVT	Ethicon, Johnson & Johnson
				Bard Mesh	Bard/Davol
				SPARC	American Medical Systems
				In-Fast	American Medical Systems
				Monarc	American Medical Systems
				Lynx	Boston Scientific
				Advantage	Boston Scientific
				Obtryx	Boston Scientific
				Optilene	B. Braun
				Aris	Mentor Corp
				Perigee	American Medical Systems
				Parietene	Covidien
				Intepro	American Medical Systems
				Gynecare Prolift	Ethicon, Johnson & Johnson
				Surgipro	Covidien
				Prolene	Ethicon, Johnson & Johnson
				Prolene Soft	Ethicon, Johnson & Johnson
				Gynemesh PS	Ethicon, Johnson & Johnson
				Atrium	Atrium Medical
				Marlex	C. R. Bard
		Multifilament	Copolymer of glycolide (90%) and lactide (10%)	Vicryl	Ethicon, Johnson &Johnson
			Polypropylene and polyglecaprone	Vypro	Ethicon, Johnson & Johnson
				UltraPro	Ethicon, Johnson & Johnson
			Polyglycolic acid	Dexon	Davis and Geck

II	Macroporous <10 *μ*m	Multifilament	Expanded PTFF	GORE-TEX	W. L. Gore
			Polyethylene terephthalate	Mersuture	Ethicon, Johnson & Johnson

III	Macroporous with microporous components <10 *μ*m	Multifilament	PTFE	Teflon	C. R. Bard
			Polyethylene terephthalate	Mersilene	Ethicon, Johnson & Johnson
			Polypropylene	IVS Tunneller	Tyco Healthcare
			Woven polyester	Protegen	Boston Scientific

IV	Nanoporous <1 *μ*m	Multifilament	Silicon-coated polyester	Intermesh	American Medical Systems
			Dura mater substitute	PRECLUDE MVP Dura substitute	W. L. Gore
			Expanded PTFE, pericardial membrane substitute	PRECLUDE Pericardial Membrane	W. L. Gore

**Table 2:** Autologous fascia.

Author	Sample	Biomechanical properties	Host response
FitzGerald et al., 2000 [23]	Autologous rectus fascia implanted in 5 patients suffering from SUI. Samples obtained, respectively, from transvaginal revision after 3, 5, 8, and 17 weeks and from replacement after 4 years.		(i) Moderate and uniform infiltration of host fibroblasts and neovascularization after 5 and 8 weeks of implantation. (ii) After 4 years of implantation, no evidence of inflammatory cell infiltrate or foreign body reaction and collagen remodeling by connective tissue organized longitudinally.

Jeong et al., 2000 [24]	Autologous lata fascia implanted in 16 rabbits randomized into 4 survival groups and examined after 1, 2, 4, and 8 weeks. Implantation into upper eyelids.		(i) Low inflammatory cell infiltration. (ii) Fibroblast infiltration and collagen remodeling.

Choe et al., 2001 [21]	Dermis, rectus fascia, and vaginal mucosa harvested from 20 women undergoing vagina prolapse surgery.	Tensiometric analysis of full strips versus patch suture slings. Displacement and maximum load calculated.	

Kim et al., 2001 [22]	Autologous rectus fascia implanted in 20 rats randomized into 2 survival groups (2 and 4 months).	No significant decrease of the fracture toughness calculated by the trouser tear test over 4 months.	

Dora et al., 2004 [19]	Autologous rectus fascia implanted in 15 rabbits randomized into 3 survival groups (2, 6, and 12 weeks). Implantation on the anterior rectus fascia.	No significant decrease of biomechanical properties after 12 weeks of implantation.	50% decrease in surface area.

Hilger et al., 2006 [20]	Autologous rectus fascia implanted in 20 rabbits randomized into 2 survival groups (6 and 12 weeks). Half implanted on the rectus fascia and half on the posterior vagina fascia.	No significant decrease of biomechanical properties after 12 weeks of implantation.	(i) Collagen remodeling by moderate collagen infiltration but encapsulation as well. (ii) Minimal inflammatory response. (iii) Minimal neovascularization.

Krambeck et al., 2006 [26]	Autologous rectus fascia implanted subcutaneously on the anterior rectus fascia of 10 rabbits randomized into 2 survival groups (6 and 12 weeks).		(i) Moderate fibrosis. (ii) High degree of scarring. (iii) High degree of inflammatory infiltrate.

de Almeida et al., 2007 [29]	Adult female rats incontinence model. Marlex, autologous sling, SIS, polypropylene mesh, and sham at 30 and 60 days.		Reduced inflammatory response and collagen production around autologous grafts, in comparison with synthetic materials and xenografts.

Woodruff et al., 2008 [27]	Autologous fascia grafts explanted after sling revision from 5 women, due to different complications, between 2 and 65 months after implantation.		(i) Moderate and uniform infiltration of host fibroblasts and little neovascularization. (ii) Collagen remodeling by new collagen fibers organized longitudinally. (iii) No evidence of encapsulation or gross infection.

de Rezende Pinna et al., 2011 [28]	Autologous fascia lata implanted in 14 rabbits randomized into 2 survival groups (30 and 60 days). Implantation into the right voice muscle.		(i) No significant inflammatory reaction. (ii) No significant fibrosis or scarring.

**Table 3:** Allografts.

Author	Sample	Biomechanical properties	Host response
Sclafani et al., 2000 [37]	Human cadaveric dermis (AlloDerm) disk implanted subdermally behind a patient's ear. Micronized human cadaveric dermis (AlloDerm) injected intradermally and subdermally in 2 different locations behind a patient's ear. Both implants were examined 3 months and 1 month after implantation, respectively.		(i) Both materials extensively invaded by host fibroblasts. (ii) Both materials present new collagen ingrowth.

Kim et al., 2001 [22]	Human cadaveric fascia implanted in 20 rats randomized into 2 survival groups (2 and 4 months).	No significant decrease of the fracture toughness calculated by the trouser tear test.	

Walter et al., 2003 [34]	Freeze-dried and gamma-irradiated human cadaveric lata fascia implanted in 18 rabbits and excised 12 weeks after implantation.	Significant decrease of biomechanical properties after 12 weeks of implantation.	

Spiess et al., 2004 [35]	Human cadaveric fascia lata implanted subcutaneously on the abdominal wall of 20 rats randomized into 2 survival groups (6 and 12 weeks).	No significant decrease of tensile strength with time.	

Yildirim et al., 2005 [38]	Human cadaveric lata fascia implanted subcutaneously on the abdominal wall in 20 rabbits randomized into 4 survival groups (2, 7, 15, and 30 days).		(i) Acute inflammation by high cell infiltration predominantly of polymorphous granulocytes. (ii) Integration in host tissue by moderate fibrotic process and muscle infiltration on day 30, with persistent inflammatory response.

Krambeck et al., 2006 [26]	Cadaveric fascia lata implanted subcutaneously on the anterior rectus fascia of 10 rabbits randomized into 2 survival groups (6 and 12 weeks).		(i) Moderate to high focal fibrosis. (ii) Minimal to moderate degree of scar. (iii) High degree of inflammatory infiltrate.

Hilger et al., 2006 [20]	Human cadaveric dermis and lata fascia implanted in 20 rabbits randomized into 2 survival groups (6 and 12 weeks). Half implanted on the rectus fascia and half on the posterior vagina fascia.	Very significant decrease of biomechanical properties after 12 weeks of implantation.	(i) Two missing or fragmented materials implanted on the vagina after 12 weeks. (ii) Moderate inflammatory response. (iii) Minimal neovascularization. (iv) Minimal collagen ingrowth without significant cell infiltration.

Woodruff et al., 2008 [27]	Human cadaveric dermis slings explanted after revision from 2 women, due to different complications, between 2 and 65 months after implantation.		(i) Moderate levels of encapsulation. (ii) High levels of degradation. (iii) Peripheries of the grafts invaded by fibroblasts but central portions remained acellular.

VandeVord et al., 2010 [39]	Human cadaveric dermis and fascia lata implanted in 16 rats, respectively, and both randomized into 4 survival groups (2, 4, 8, and 12 weeks). Implantation around the bladder neck, anchored to the surrounding tissues.		(i) Thin fibrous capsule formation. (ii) Moderate cell infiltration and angiogenesis.

Rice et al., 2010 [36]	Human cadaveric dermis (AlloDerm) implanted in 18 rats randomized into 2 survival groups (30 and 60 days). Subcutaneous implantation on abdominis rectus muscle defect.	Increase of tensile strength after 30 days and, again, increase of tensile strength after 60 days, respectively, to 30 days.	(i) Moderate amounts of collagen deposition well organized. (ii) Abundant revascularization.

Kolb et al., 2012 [40]	Human cadaveric dermis (AlloDerm) implanted subcutaneously in 5 pigs randomized into 4 survival groups (7, 21, 90, and 180 days).		(i) Robust inflammatory response after 7 days of implantation, which achieved maximal level at 21 days, with formation of granulomas and areas of necrosis noted within the graft. (ii) Moderate fibroblast infiltration, collagen ingrowth, and neovascularisation. (iii) Moderate levels of encapsulation.

**Table 4:** Xenografts.

Author	Sample	Biomechanical properties	Host response
Badylak et al., 2001 [52]	Abdominal wall defect repaired with SIS in 40 dogs randomized into 8 survival groups (1, 4, 7, and 10 days and 1, 3, 6, and 24 months).	Strength was decreased from day 1 to day 10 after implantation, followed by a progressive increase, until reaching double of the original strength 24 months after implantation.	Rapid degradation with associated and subsequent host remodeling.

Badylak et al., 2002 [55]	Abdominal wall defect repaired with SIS in 10 dogs and 30 rats, both randomized into 4 survival groups (1 week, 1 month, 3 months, 6 months, and 2 years).		(i) No shrinkage or expansion of the graft site over the 2-year period of the study. (ii) One week after implantation, abundant levels of polymorphonuclear leukocytes diminished to negligible after 1 month. (iii) Moderate neovascularization. (iv) By 3 months, graft material was not recognizable and was replaced by moderately well-organized host tissues including collagenous connective tissue, adipose tissue, and skeletal muscle.

Cole et al., 2003 [60]	SIS removed from a 42-year-old female patient 4 months after pubovaginal implantation of the sling due to severe obstruction.		(i) Completely intact acellular sling. (ii) Well defined fibrous capsule. (iii) Chronic inflammatory response.

Zhang et al., 2003 [51]	SIS implanted in the abdominal wall of rats for up to 2 months.	SIS together with the abdominal wall has increased strength.	Levels of interleukin 2 and interleukin 6 were high straight after the operation but they become normal after 2 months.

Wiedemann and Otto, 2004 [56]	Biopsies taken from the implantation site of the SIS band under the vaginal mucosa from 3 patients during reoperation, at a mean of 12.7 months, after pubourethral sling procedures due to recurrent urinary stress incontinence.		(i) Focal residues of SIS implant. (ii) No evidence of a specific tissue reaction that might point to a foreign body reaction. (iii) No evidence of any significant immunological reaction and in particular no evidence of any chronic inflammatory reaction.

Konstantinovic et al., 2005 [50]	Abdominal wall defect repaired with SIS in 24 Wistar rats randomized into 4 survival groups (7, 14, 30, and 90 days).	Significant increase of biomechanical properties after 90 days of implantation.	(i) Moderate acute inflammatory response at day 7, decreased to minimal after 90 days. (ii) Moderate neovascularization. (iii) Abundant collagen deposition well organized after 90 days.

Macleod et al., 2005 [62]	SIS and cross-linked porcine dermis (Permacol) implanted subcutaneously on the anterior rectus fascia of 18 rats each randomized into 5 survival groups (1, 2, 4, 10, and 20 weeks).		For both grafts: (i) absent acute inflammatory response, (ii) from moderate chronic inflammation after 1 week of implantation to minimal after 20 weeks, (iii) absent eosinophilic infiltration and stromal fibroblastic reaction over the entire implantation, (iv) from moderate fibrosis and vascularity around the grafts after 1 week of implantation to minimal after 20 weeks.

Poulose et al., 2005 [57]	12 female pigs were implanted with SIS intraperitoneally for up to 6 weeks.		(i) Cell infiltration. (ii) Vascularization. (iii) Collagen deposition and remodelling.

Thiel et al., 2005 [58]	SIS implanted subcutaneously on the abdominal wall of 30 rats randomized into 3 survival groups (7, 30, and 90 days).		(i) Moderate inflammatory reaction increased to severe after 90 days. (ii) 86% of the graft was replaced by new collagen fibers.

Krambeck et al., 2006 [26]	SIS and porcine dermis implanted subcutaneously on the anterior rectus fascia of 10 rabbits randomized into 2 survival groups (6 and 12 weeks).		(i) Porcine dermis presented moderate fibrosis which was minimal for SIS. (ii) Minimal degree of scar for both grafts and high degree of inflammatory infiltrate.

Ko et al., 2006 [54]	Abdominal wall defect repaired with 8-layer SIS in 20 domestic pigs randomized into 2 survival groups (1 and 4 months).	No significant changes of biomechanical properties after 4 months of implantation.	(i) Dense fibrous connective tissue ingrowth. (ii) Minimal to mild mononuclear inflammatory cell infiltrate throughout the connective tissue.

Hilger et al., 2006 [20]	Porcine dermis implanted in 20 rabbits randomized into 2 survival groups (6 and 12 weeks). Half implanted on the rectus fascia and half on the posterior vagina fascia.	Very significant decrease of biomechanical properties after 12 weeks of implantation.	(i) Two missing or fragmented materials 12 weeks after being implanted on the vagina. (ii) Moderate to strong inflammatory response. (iii) Minimal collagen ingrowth without significant cell infiltration. (iv) Minimal neovascularization.

Kim et al., 2007 [59]	SIS implanted in the subcutaneous dorsum of 3 rats sacrificed after 2 weeks.		(i) Prominent infiltration and ingrowth of host cells. (ii) Few macrophages infiltrated or accumulated around the grafts.

Rauth et al., 2007 [63]	SIS implanted on the peritoneal surface of the abdominal wall of 6 pigs sacrificed 8 weeks after implantation.		(i) 80% of contraction from original surface area. (ii) Moderate neovascularization. (iii) Densely populated by host cells with moderate amounts of new disorganized collagen deposition.

Woodruff et al., 2008 [27]	Porcine dermis slings explanted after revision from 4 women, due to different complications, between 2 and 65 months after implantation.		(i) Severe encapsulation. (ii) No degradation. (iii) No fibroblasts infiltration and neovascularization.

Sandor et al., 2008 [64]	Abdominal wall defect repaired with SIS and cross-linked porcine dermis (Permacol) in 33 primates randomized into 3 survival groups (1, 3, and 6 months).		(i) Considerable contraction after 1 month for both materials, but not significant change over the next 5 months. (ii) Better integration of both materials at late stage by scar formation. (iii) Inflammatory cells infiltration 3 months after implantation for SIS associated with formation of few blood vessels. (iv) Acellular porcine dermis over the entire course implantation with substantial inflammation surrounding their perimeter. (v) Partial resorption for both materials after 6 months.

Pierce et al., 2009 [65]	Cross-linked porcine dermis implanted on the abdominal wall and posterior vagina of 18 rabbits sacrificed 9 months after implantation.	11 grafts remained intact without significant changes of biomechanical properties compared to the baseline values. They were just thicker and tolerated with less elongation at failure. Seven grafts were partially degraded but thicker again and with significant decrease of all biomechanical properties.	(i) Host connective tissue incorporation between fibers. (ii) Intense foreign body reaction in degraded grafts.

VandeVord et al., 2010 [39]	SIS and porcine dermis implanted in 16 rats, respectively, and both randomized into 4 survival groups (2, 4, 8, and 12 weeks). Implantation around the bladder neck, anchored to the surrounding tissues.		(i) Thin fibrous capsule formation. (ii) Moderate cell infiltration and angiogenesis for SIS and minimal for porcine dermis.

Rice et al., 2010 [36]	Abdominal wall defect repair with SIS (Surgisis) in 18 rats randomized into 2 survival groups (30 and 60 days).	Increase of tensile strength after 30 days and, increase of tensile strength after 60 days, respectively, to 30 days.	(i) Moderate amounts of collagen deposition well organized. (ii) Abundant revascularization.

Deprest et al., 2010 [61]	13 patients underwent secondary sacrocolpopexy because of a graft related complication after the initial sacrocolpopexy with porcine dermal collagen (Pelvicol) (9) or SIS (Surgisis) (4).		(i) Pelvicol presented high degradation rates associated with no foreign body reaction. (ii) Pelvicol remnants were integrated into collagen rich connective tissue with limited neovascularization (scar host tissue). (iii) No significant body foreign reaction to Surgisis grafts. (iv) Surgisis no longer recognizable replaced by irregularly organized connective tissue and fat tissue.

Liu et al., 2011 [49]	Abdominal wall defect repaired with SIS and acellular porcine dermal matrix in 50 Sprague Dawley rats randomized into 5 survival groups (1, 2, 4, 8, and 12 weeks).	After initial decrease of biomechanical properties at week 2, these were increased over the next 10 weeks reaching similar values to week 1.	(i) Pronounced inflammatory response 1 to 4 weeks after implantation for SIS compared with porcine dermis, but falling to similar negligible values for both after 12 weeks. (ii) Large neovascularization and collagen deposition, which was higher for SIS group. (iii) SIS implants degraded more quickly and were almost totally replaced by organized collagenous tissues. (iv) Contraction at the first weeks leading to significant lower surface area in both materials.

Jenkins et al., 2011 [53]	Abdominal wall defect repaired with porcine dermal matrix in 24 Yucatan minipigs randomized into 2 survival groups (1 and 6 months).	Significantly greater incorporation strengths after 6 months compared with 1 month.	(i) Moderate cell infiltration. (ii) Moderate extracellular matrix deposition. (iii) Moderate neovascularisation. (iv) Partial degradation and from widely to mild fibrous encapsulation.

Kolb et al., 2012 [40]	Cross-linked porcine dermis (Permacol) implanted subcutaneously in 5 pigs randomized into 4 survival groups (7, 21, 90, and 180 days).		(i) Mild inflammatory response decreased to minimal from day 7 to day 180 after implantation. (ii) None to minimal neovascularization after 180 days. (iii) Small amount of residual SIS remained surrounded by mild to moderate chronic inflammation. (iv) Moderate levels of encapsulation.

Daly et al., 2012 [66]	Abdominal wall defect repaired with porcine dermis in rats randomized into 3 survival groups (1, 3, and 35 days).		(i) Cell infiltrates into all grafts by day 35. (ii) Degradation of the scaffold most pronounced at the periphery with fibrous tissue, angiogenesis, and foreign body giant cells noted. (iii) Grafts surrounded by a dense and circumferentially organized connective tissue. (iv) Mononuclear cells decreased in number compared with earlier time points.

**Table 5:** Polypropylene meshes.

Author	Sample	Biomechanical properties	Host response
Falconer et al., 2001 [89]	16 women were implanted with TVT for up to 2 years: 6 with Mersilene and 10 with Prolene.		Mersilene induces higher inflammatory response than Prolene. Mersilene is easier to extract than Prolene.

Klinge et al., 2002 [80]	Heavy weight monofilament with small pore size (HWM) and low weight with large pore size multifilament (LWM) on the posterior abdominal wall of rats for 7, 14, 21, and 90 days.		(i) HWM: intense inflammation, embedded in connective tissue. (ii) LWM: less pronounced inflammatory response and fibrotic capsule, with collagen distributed within the mesh.

Wang et al., 2004 [90]	17 women with sling erosion and 7 women with voiding difficulties implanted with TVT and SPARC.		Pronounced fibrosis around the fibers—erosion and voiding difficulty as a result.

Rabah et al., 2004 [84]	Implantation of Surgipro and cadaveric fascia lata in rabbit's bladder neck for 6 and 12 weeks.		(i) Cadaveric fascia lata group: the implant was incorporated in a plate of fibrous tissue. (ii) Polypropylene mesh: inflammation localized on the graft.

Spiess et al., 2004 [35]	TVT and cadaveric fascia lata implanted in abdominal wall of rats for 6 and 12 weeks.	TVT has the greater break load and the maximum average load compared to cadaveric fascia lata.	

Zheng et al., 2004 [81]	Prolene and Pelvicol implanted in full thickness abdominal wall defects in rats for 7, 14, 30, and 90 days.		Prolene prosthesis shows the presence of leukocytes in the activated state.

Konstantinovic et al., 2005 [50]	Marlex and non-cross-linked Surgisis implanted on the anterior abdominal wall of rats for 7, 14, 30, and 90 days.		(i) Marlex: more pronounced inflammatory reaction and vascularization throughout the graft than Surgisis (ii) Surgisis: milder inflammatory reaction.

Yildirim et al., 2005 [38]	Gynecare TVT, SPARC, polypropylene mesh, and IVS implanted in contact with the rats rectus muscle for up to 30 days.		Inflammation and fibrosis are decreased in large pore meshes.

Thiel et al., 2005 [58]	Monofilament polypropylene mesh, silicone mesh, SIS, and PLA were implanted subcutaneously on the abdomen of rats for 7, 30, and 90 days.		Polypropylene induces the mildest inflammatory response among the samples.

Bogusiewicz et al., 2006 [83]	Monofilament TVT and multifilament IVS were implanted in rats rectus fascia for 42 days.		(i) They induce production of similar amount of collagen. (ii) Differences in the arrangement of collagen and inflammation intensity.

Boulanger et al., 2006 [87]	Vicryl, Vypro, Prolene, Prolene Soft, and Mersuture were implanted in pigs peritoneum for 10 weeks.		(i) Vicryl: low level of inflammation and completely absorbed. (ii) Vypro: intense inflammation and strong fibrotic response. (iii) Prolene and Prolene Soft: well integrated, weak inflammatory response. (iv) Mersuture: no good integration.

Krambeck et al., 2006 [26]	SPARC mesh, human cadaveric fascia, porcine dermis, SIS, and autologous fascia were implanted in rabbits rectus fascia for 12 weeks.		(i) Polypropylene mesh has the greatest scar formation. (ii) Polypropylene has the mildest inflammatory response.

Boukerrou et al., 2007 [75]	Preperitoneal implantation of Vicryl, Vypro, Prolene, Prolene Soft, and Mersuture mesh for 2 months in pigs.	Nonabsorbable, monofilamentous, macroporous materials (type I) seem more resistant, retract less, and have the best tolerance.	.

Spelzini et al., 2007 [82]	Polypropylene type I mesh and macroporous silk construct were implanted in rat fascial defects for 7, 14, 30, and 90 days.		Polypropylene meshes induce a moderate inflammatory response and not architectural degradation.

Zorn et al., 2007 [74]	Rat abdominal wall was implanted with SPARC, TVT, and SIS for 6 weeks and 9, 6, 9, and 12 months.	TVT has tensile properties similar to SPARC and they are superior to Stratasis.	

Bazi et al., 2007 [76]	Rats rectus fascia was implanted with Advantage, IVS, SPARC, and TVT for up to 24 weeks.	They all show similar mechanical properties after removal.	They induce different host responses due to different porosity.

Tayrac et al., 2007	Ewes vaginas were implanted with a noncoated LW polypropylene mesh (Soft Prolene) and a coated one (Ugytex) from 1 to 12 weeks.		Similar inflammatory response between the two materials.

Huffaker et al., 2008 [86]	Rabbits vaginas were implanted with Pelvitex (collagen-coated) and Gynemesh (uncoated polypropylene meshes) for up to 12 weeks.		Both materials induce a mild foreign body reaction with minimal fibrosis.

Woodruff et al., 2008 [27]	24 grafts were explanted in women undergoing sling revision after 2–34 months. Grafts were polypropylene meshes, autologous fascia, porcine dermis, and cadaveric dermis.		No evidence of degradation or encapsulation, abundant host infiltration. Neovascularisation was visible.

Elmer et al., 2009 [91]	Prolift was implanted in humans for 1 year.		(i) Increase in macrophages and mast cells count. (ii) Mild but persistent foreign body response.

Pierce et al., 2009 [65]	Polypropylene mesh versus cross-linked porcine dermis implanted in rabbits vagina and abdomen for 9 months.		Polypropylene caused milder inflammatory reaction, more long term, good host tissue incorporation.

Melman et al., 2011 [77]	Bard mesh (HWPP), Ultrapro (LWPP), and GORE INFINIT Mesh (ePTFE) in minipigs hernia repair for 1, 3, and 5 months.	Their maximum tensile strength decreases for all of them.	(i) Inflammation decreases with time. (ii) Cell infiltration increases with time.

Pascual et al., 2012 [85]	Surgipro, Optilene, and GORE INFINIT Mesh (ePTFE) were implanted in rabbits abdominal wall defect for 14 days.	LWPP implants might be improved by the newly formed tissue around it.	(i) PTFE induces an increased macrophage response when compared to polypropylene. (ii) Increase in collagen deposition in high porosity meshes.

Manodoro et al., 2013 [78]	Gynemesh in two sizes (50 × 50 mm and 35 × 35 mm) implanted in 20 adult ewes for 60 and 90 days, both on the abdominal and vaginal walls.	Implants were contracting more when implanted on the vaginal wall, compared to abdominal wall. Grafts implanted on the vaginal wall are stiffer than the ones implanted on the abdominal wall, after retrieval.	(i) 30% of the 50 × 50 meshes caused vaginal erosion and exposure. (ii) 60% of the 35 × 35 meshes had reduced surface (i.e., contracting after 90 days.)

HWPP: heavy weight polypropylene.

LWPP: lightweight polypropylene (also called *soft*); ePTFE: expanded polytetrafluoroethylene; PLGA: polylactide-co-glycolide acid; PLA: polylactide acid; PGA: polyglycolide acid.
